# Relational aspects of building capacity in economic evaluation in an Australian Primary Health Network using an embedded researcher approach

**DOI:** 10.1186/s12913-022-08208-7

**Published:** 2022-06-22

**Authors:** Donella Piper, Christine Jorm, Rick Iedema, Nicholas Goodwin, Andrew Searles, Lisa McFayden

**Affiliations:** 1 NSW Regional Health Partners, Newcastle, Australia; 2 School of Medicine and Public Health, University of Newcastle, Newcastle, Australia; 3 School of Public Health, University of Sydney, Sydney, Australia; 4 Centre for Team-Based Practice & Learning in Health Care, King’s College, London, UK; 5 Central Coast Research Institute for Integrated Care, University of Newcastle & Central Coast Local Health District, Newcastle, Australia; 6 Health Research Economics, Hunter Medical Research Institute, Newcastle, Australia; 7 University of Newcastle, Newcastle, Australia

**Keywords:** Health economics, Economic evaluation, Program evaluation, Embedded researcher, Health services research, Value-based healthcare, Primary care, Commissioning, Australia

## Abstract

**Background:**

Health organisations are increasingly implementing ‘embedded researcher’ models to translate research into practice. This paper examines the impact of an embedded researcher model known as the embedded Economist (eE) Program that was implemented in an Australian Primary Health Network (PHN) located in regional New South Wales, Australia. The site, participants, program aims and design are described. Insights into the facilitators, challenges and barriers to the integration of economic evaluation perspectives into the work of the PHN are provided.

**Methods:**

The eE Program consisted of embedding a lead health economist on site, supported by offsite economists, part-time, for fifteen weeks to collaborate with PHN staff. Evaluation of the eE at the PHN included qualitative data collection via semi-structured interviews (*N*= 34), observations (*N*=8) and a field diary kept by the embedded economists. A thematic analysis was undertaken through the triangulation of this data.

**Results:**

The eE Program successfully met its aims of increasing PHN staff awareness of the value of economic evaluation principles in decision-making and their capacity to access and apply these principles. There was also evidence that the program resulted in PHN staff applying economic evaluations when commissioning service providers. Evaluation of the eE identified two key facilitators for achieving these results. First, a highly receptive organisational context characterised by a work ethic, and site processes and procedures that were dedicated to improvement. Second was the development of trusted relationships between the embedded economist and PHN staff that was enabled through: the commitment of the economist to bi-directional learning; facilitating access to economic tools and techniques; personality traits (likeable and enthusiastic); and because the eE provided ongoing support for PHN projects beyond the fifteen-week embedding period.

**Conclusions:**

This study provides the first detailed case description of an embedded health economics program. The results demonstrate how the process, context and relational factors of engaging and embedding the support of a health economist works and why. The findings reinforce international evidence in this area and are of practical utility to the future deployment of such programs.

**Supplementary Information:**

The online version contains supplementary material available at 10.1186/s12913-022-08208-7.

## Introduction

Decisions are made daily in healthcare about the type of care that will be provided, not only to individual patients but also to improve people’s overall health and wellbeing. These decisions can be about new medicines, new technologies, improved models of care or approaches designed to promote health and prevent or manage avoidable illnesses within the population [1].

Determining whether healthcare spending choices represent value - for governments, patients and taxpayers - depends on whose perspective is taken, but in general, ‘value’ can be assessed by examining the impacts of spending choices on health service efficiency and equity. While such evaluation is common at the national level, economic evaluations are relatively rare at the local level [2]. The local level includes local health districts, hospitals, community and primary care services and it is where Australia spends most of its health budget. There are a multitude of reasons why economic evaluations are not done at the local level, but key ones are a lack of health economic skills in the health workforce and/or a lack of access to appropriately experienced health economists [2].

The gap in health economic skills in local health services was an important finding in a national report on local level evaluation [2]. Insights from this work showed local health services wanted to develop their own internal capacity and capability in evaluation, particularly in health economics [2]. They also wanted to work with experts who would be focused on the *priorities of the health service* and share their skills with staff, a focus that was often lacking when private sector or academic expertise was engaged [2]. The embedded Economist (eE) Program was developed to address this need by embedding a health economist in health services to build capacity in economic evaluation. The embedding was undertaken over approximately three months each in four local health districts (LHDs) and also in a Primary Health Network (PHN). This paper reports the evaluation of the eE in the Hunter New England Central Coast Primary Health Network (HNECCPHN).

The paper firstly presents the distinct PHN context and the background that informed the ‘economist-in-residence’ approach. Following sections present methods, findings and impact of the eE Program, before distilling our study results into a more general argument in support of developing local capacity and capability in economic evaluation.

## Background

### Australian Primary Health Networks

Australian PHNs were established on 1^st^ July 2015 by the Australian Federal Government to increase the effectiveness of medical services for patients, particularly those at risk of poor health outcomes, and to improve coordination of care. The PHNs are not-for-profit organisations which do not directly provide health services. Instead, they use a commissioning model to work with primary health care providers, secondary care providers and hospitals. Commissioning includes a range of activities to assess the needs of the population, plan and prioritise services, purchase those services and monitor the quality of the services being provided. PHN commissioning is intended to move the local health system towards more sustainable models of care by not only procuring new or additional services but also transforming and reorganising existing services [3–5].

As a ‘newcomer’ to the Australian health services landscape, there is little research overall on PHNs and the effectiveness of their commissioning [3–5]. Recent evaluations have concluded that they are ‘making progress’ in demonstrating a deeper understanding of the needs of their local communities, building partnerships to address shared priorities, and developing innovative ways to commission services [6]. However, what we know from international experience is that effective commissioning relies on evidence-informed planning and evaluation of cost against effectiveness, including valid and feasible measures of quality and/or outcomes [7, 8]. Where commissioning services have been extensively studied, such as in England, the decision-making process has been described as pragmatic, involving ‘juggling competing agendas, priorities, power relationships, demands and personal inclinations to build a persuasive, compelling case,’ necessitating a change in approach by researchers to one that ensures the provision of localised and useful information [7]. As a result, commissioning in England as an approach to improve and integrate care services has often been described as ‘weak’ due to the lack of advanced commissioning skills [9]. Attempts in England to address this skills gap have included workshops on systematic evidence searching and critical appraisal of evidence quality [10]. There has also been advocacy for the use of economic modelling tools [11], especially to improve procurement processes and contract management.

### Embedded researchers

Embedded researchers, or researchers-in-residence, are researchers who work as part of an operational team. Their role is not simply to bring new skills and expertise to the team. They are charged with ‘mobilising knowledge and creating new evidence for local use and wider dissemination’ [12]. Embedded researchers are required to ‘negotiate their expertise, integrate it with the expertise of their colleagues’ and, where necessary, compromise to reach ‘shared understanding and solutions’ [12].

This approach has been implemented in areas including education, the law, and social care [13]. Researchers have embedded across a wide variety of clinical contexts in the United Kingdom [12, 14, 15]; including public, private and voluntary settings that either commission and/or deliver primary, community and/or acute care [12, 14, 15]. Similar initiatives have been implemented in the United States and Canada [16]. There is a small but growing number of embedded researcher initiatives in Australia, most of which have taken the form of a senior academic with clinical experience collaborating with health service staff on co-producing traditional research outputs [17, 18].

Regardless of jurisdiction or clinical setting, the literature on embedded researchers is rated by Ward and colleagues [15] as lacking in analysis ‘disaggregating the components’ of such initiatives, concentrating instead on ‘overviews of the principles of embedded research.’ The result is little information about what embedded researcher initiatives look like in practice [15]. There is agreement on the need for more research into how the models are implemented, how they work, under what conditions and for whom [17–20]. Varallyay et al. [21] regard this as ‘…particularly important given the lack of clarity about the core features and conditions of “embeddedness” minimally required to achieve the objective of evidence-informed decision-making for health programme improvement.’

Despite the lack of formal evaluations to date, guidance on designing embedded initiatives is not lacking. The literature identifies and lists a number of key success factors for embedded research including various *process* [13, 18, 19, 22–25], *contextual* [18, 19, 22, 23, 26] and *relational* factors [13, 19, 22, 25, 27].

### The embedded Economist (eE) program

Against this background, this article now sets out results from embedding a health economist in the first of six program sites, a regional New South Wales (NSW) PHN. This site was unique – the others being large health services centred on hospitals. The full planned program protocol, including a brief literature review and overview has been reported in an earlier edition of this journal [1]. The conceptual underpinnings are also reported elsewhere in detail [28]. The basis of the project is ‘slow science’ [29] and focus achieving impact in the site of research, with academic outputs such as publication not an objective (but to be supported if desired by the site).

By way of summary, the eE Program had three aims:

1. To increase health service staff awareness of the value economic evaluation can bring to decision-making.
2. To develop health service staff knowledge and capacity to access and apply economic evaluation principles, methods and tools in decision-making through formal training and extended exposure to an embedded economist.
3. To facilitate health service practice change and the routine application of economic evaluation principles in decision-making [1].

The aims of the evaluation were to capture the outcomes and impact of embedding an economist and to evaluate the contextual, procedural and relational aspects that facilitated or acted as a barrier to the program [1]. More detail is provided on the relational findings as they add considerably to the published literature.

## Methods

### Setting

**Fig. 1:**
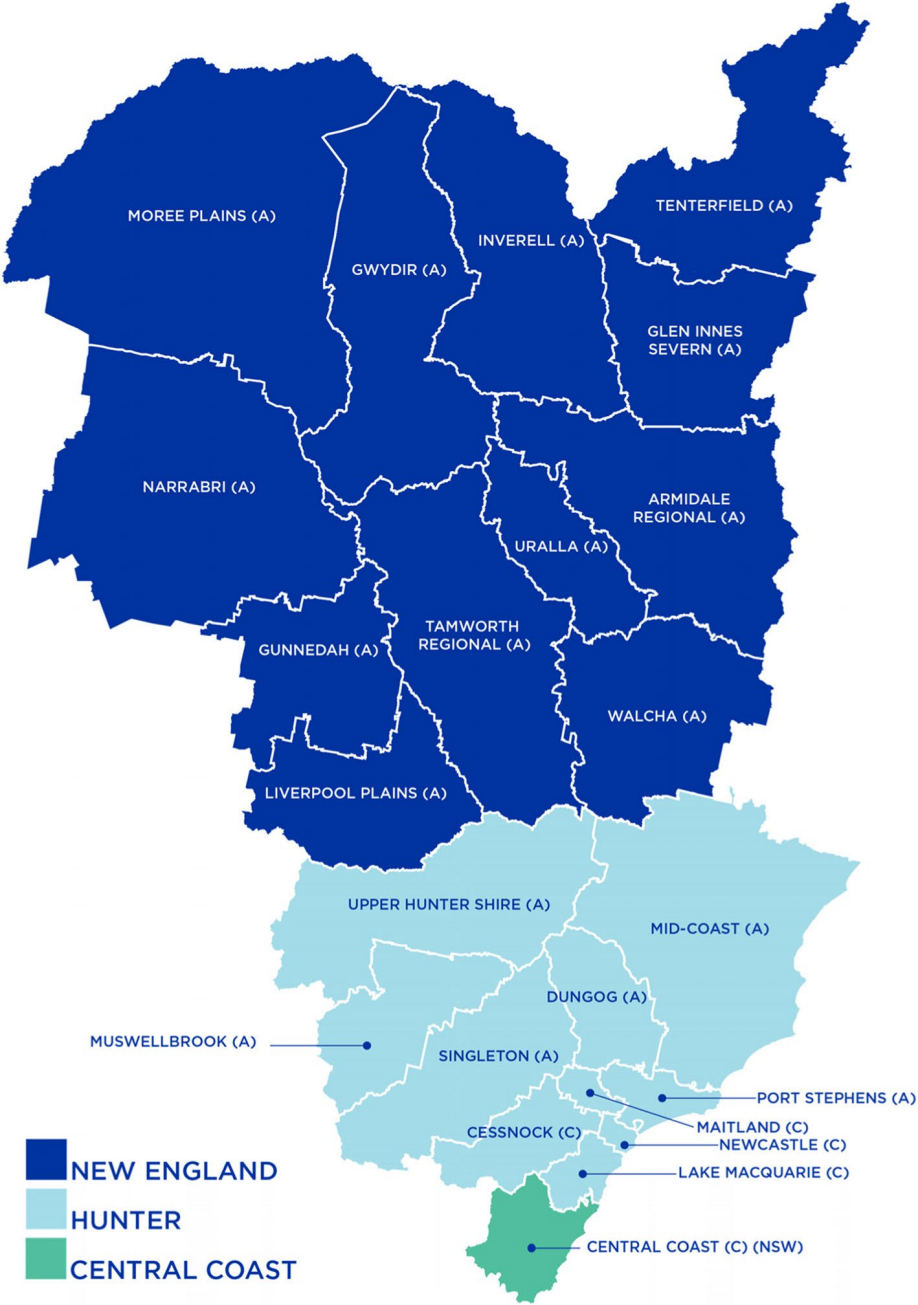
The HNECCPHN geographical footprint, Source: https://thephn.com.au/about-us

The Hunter New England Central Coast Primary Health Network (HNECCPHN) is a new (approximately six years old) and medium-sized organisation, consisting of a chief executive; four senior executives; 11 board members; a clinical council; a community council; and approximately 100 full-time equivalent staff. The HNECCPHN region covers 130,000 square kilometres, as set out in Fig. 1, and has a population of 1.2 million people who live in small rural and remote villages, in regional towns and in densely populated urban centres. The organization has three main offices in Erina, Newcastle and Tamworth, located hundreds of kilometres apart.

### Participants

The chief executive, all senior executives (*N*=4) and all staff employees (*N*=100) had some form of interaction with the eE over the three program phases. A lead health economist and four support health economists from the Hunter Medical Research Institute (HMRI) also participated. Specific details including number and type of participants are set in Supplementary file 1.

### Site study design

A senior ‘front of house’ lead health economist (professorial level) was embedded within the HNECCPHN from 15 October 2019 through to 29 February 2020. Allowing for Christmas and New Year breaks, the elapsed time was approximately fifteen weeks. The economist worked with PHN staff to conduct economic evaluations and advise on evaluation and impact design and the inclusion of economics principles, as appropriate, in day-to-day business. While the lead economist conducted all the face-to-face interactions with PHN staff, ‘back of house’ support economists were engaged from the HMRI Health Economics team to provide assistance in conducting the economic evaluations and to provide specialist expertise as needed. The program was structured into three phases: planning, embedding and post-embedding [1]. A detailed description of each phase, how they were implemented, and who participated in the HNECCPHN eE Program is set out in Supplementary file 1. Specific details about the number and type of engagement with the economist are set out in Supplementary file 2.

### Evaluation design

The evaluation took place concurrently with the eE Program, with the last interviews conducted in mid-July 2020, approximately five months after the embedding phase was complete. Ethics approval was obtained from the Human Research Ethics Committee of the University of New England (approval number H-2018-0005), with written consent provided by all participants. All methods were carried out in accordance with relevant guidelines and regulations.

Potential evaluation participants were all HMRI economists and PHN senior executives and staff members participating in the eE Program. Complementary data was collected at each phase of the project to capture insights into the context, processes, relational aspects and outcomes of the program [25]. Methodological triangulation was undertaken by cross-checking or comparing and contrasting the different data sources which add value to each other by illuminating different aspects of an issue and potentially explaining unexpected findings. For example, the reflective field diaries kept by the embedded economist provided data on what the embedded economists think and do [22, 25]. Interviews allowed issues identified by the evaluator to be explored in more detail with the economist, site executives and participants. Interviews were semi-structured and followed an interview guide based on the program aims and key themes from the embedded researcher literature. Observation notes enabled the social scientist to identify if what people said in interviews and field diaries matched what was done in real-time.

### Data collection and data analysis

The data that were collected prior, during and after the economist embedded at the PHN are listed in Table 1.

**Table 1:** Data collected for evaluation

PHASE	DATA COLLECTED
Planning	• Observation notes from 3 interactions between economists and PHN senior executives; • Six interviews (four with PHN senior executives and two with HMRI economists). • The economist’s field diary. • Documents including: emails between the site and economist and researchers; team and site meeting agendas and minutes; as well as the introductory PowerPoint presentation and draft eE Program operational plan.
Embedding	• Observations from five interactions between economists and participating PHN staff; • 20 interviews (two with PHN senior executives; two with the lead economist; 16 with PHN staff participants). • The economist’s field diary. • Emails between the site and economist and researchers; and research team meeting agendas and minutes.
Post-embedding (after the economist left the site)	• 12 interviews (three with PHN senior executives; one with the lead economist and two with support economists; six with PHN staff participants who were also interviewed during the embedded phase). • Emails between the site and economists and researchers; and research team meeting agendas and minutes.

Interviews were recorded with consent and professionally transcribed. Participants were invited to review their de-identified transcripts and request amendments before they are entered into QSR International’s NVivo qualitative data analysis software (Version 12) [30]. Documents were provided by the sites. The researchers also produced field diaries and researcher observations. All data were entered into NVivo.

A thematic analysis was conducted on the data by applying a coding framework using predetermined themes. The themes were based on a review of the embedded literature conducted by the researchers, used to design the program (and summarised in the introduction of this paper) as well as the program aims. The coding framework was tested and refined by two of the researchers (DP and CJ), with each blind coding six interviews and refining the manual and coding based on a comparison of their coding of these initial interviews. The refined coding manual was then applied to the remaining transcripts via an iterative, reflexive and concurrent process of refining themes [31, 32]. The coded themes were corroborated and legitimated by scrutinising the previous stages to ensure that clustered themes were representative of the initial data analysis and assigned codes.

## Results

### Key outputs

The key outputs include six projects that were undertaken by the HNECCPHN whilst the economist was embedded. A variety of economic evaluation skills and tools were developed and applied during the projects including: business case development; applying cost modelling principles and cost consequence tools and processes; developing and implementing a program logic model; and identifying and collecting appropriate outcome measures to support program evaluation and impact assessment. Supplementary file 3 presents an overview of the projects.

### Key impacts

Key impacts are summarised in Table 2. Interviews with *all* program participants suggests the program successfully increased staff awareness of the benefits of economic evaluation. Increased awareness took the form of looking at work differently and thinking about evaluation more, from a program or initiatives inception. Both senior executive (*N*=2) and staff participants (*N*=10) believed the eE Program had developed their capacity to access and apply economic evaluation. It did so by providing additional information, knowledge and tools. For example, a variety of economic evaluation skills and tools were developed and applied via the six projects including: developing a business case; applying cost modelling / cost consequence tools and processes; developing and implementing a program logic model; and identifying and collecting appropriate outcome measures to support program evaluation and impact assessment (see Supplementary file 3 for more detail on tools and skills development). In addition, interviews with the site lead and staff participants (*N*=3) suggest there was limited but emerging evidence to demonstrate practice change. However, senior executive (*N*=2) and staff participants (*N*=12) stated they wanted ongoing support if capacity building was to continue:

*Ongoing support would be ideal…what we found was we – we were sorry that the time was coming to an end because we saw an ongoing need for the embedded Economist in providing further support and upskilling, and capability development, which I suppose reflects the success of the initiative in its early stages. PHN PARTICIPANT 4*

**Table 2:** Overview of key impacts

IMPACT		QUOTE
**Increased awareness of the benefits of economic evaluation**	Overall	*The biggest outcome would be just overall improved awareness and understanding of the need and benefit of better evaluation and utilising some of those tools across the organisation. PHN PARTICIPANT 13*
	Working differently and thinking about evaluation more	*…it’s helped people to start to look at what they’re doing a little bit differently, and look past just sort of tick box, ‘did you enjoy the program?’ kind of thing, to being able to assess it more fully…the main impact is that it’s highlighted better evaluation for our programs so people are thinking about it more… and even for the executive, in particular, they’re starting to look at a bit more about program impacts and economic value versus health value. For me and my team, it is just a greater awareness of how to go about doing that and making sure that we consider that when we’re developing up or scoping up new programs and projects so that’s part of what we do, and business as usual. PHN PARTICIPANT 5*
	Inclusion of evaluation from program inception	*For every project that I am given I will definitely consider evaluation from the get-go rather than halfway through, or even at the end. PHN PARTICIPANT 10*
**Developing staff capacity to access and apply economic evaluation**	Overall	*I was hoping for an increase in knowledge and capability around health economic assessment and evaluation in the organisation, from the initiative. And from what I’ve seen so far I’m encouraged that there’s been a positive benefit in that direction… PHN PARTICIPANT 4*
	By providing additional information, knowledge and tools	*[The eE] brought with him some different information and knowledge, and ways of evaluating from an economic point of view, but also from a general point of view, that they hadn’t really thought of, and so it gave them additional tools to then have conversations about well… if we use this model of care we can improve the care for ‘X’ amount of people and that’s a saving of so many dollars, or whatever.. PHN PARTICIPANT 5*
	Example: The Medical Practice Assistant Program (MPA)	*…we've been involved in the whole process … right from the beginning we worked jointly on the program logic for the overall MPA program…workshopped what the impacts would be …, from the point of view of a range of stakeholders, GPs and registered nurses and the MPA graduates and students themselves, and what the domains of those impacts might be. Then we were jointly involved in developing the survey tools … and the interview questions for the graduates. We've all been involved in the distribution of the instruments [and] workshopping the various issues we've had along the way, in terms of how we would address low response rates, how we would incentivise people to do the survey… we haven't gotten to the cost and benefit stuff yet… PHN PARTICIPANT 18*
**Emerging evidence of change**	Overall	*I’m seeing it at my manager’s meetings, which we hold monthly. I’m also hearing it in conversations with staff on the ground when … a general practice wants to do something slightly differently. The conversations are around ‘well, what’s that going to cost, is it really worth us doing that’? SITE LEAD*
	Increased use of logic models in program planning and evaluation	*We’re starting to use some logic models which – which is helping obviously. PHN Participant 5*
	Increased consideration of the need to evaluate commissioning contracts	*I put it into my contracts with my new providers, as part of a deliverable – and [the eE] gave me permission in a way to do this… they have to submit an impact evaluation report. [And that wasn’t in there before the eE Program]? No. There was no evaluation report whatsoever PHN PARTICIPANT 8* *In meetings when I’ve got my managers in a monthly management meeting, those types of questions will come up. Or … I’ll see an email where they’re pitching something … Whereas normally their argument … could be about, ‘Well, this is how we’ve always done it,’ or, ‘This is government policy’. Or ‘health has funded us to do this.’ While all those things are still really important, they’re now adding in that other element. ‘Is it really the best way of doing it?’ ‘Could we do an evaluation?’ ‘Could we spend this $20,000 on an evaluation, rather than just rolling it over again this year?” That’s the sort of stuff that I’m getting, which is really great. That’s really great. SITE LEAD*

### Key findings

Tables 3, 4, and 5 set out the process, contextual and relational facilitators and barriers encountered for each phase of the program.

**Table 3:** Summary of planning phase process facilitators

FACILITATORS	QUOTE
**Board and executive sponsorship**	*Our Board are aware of us doing this, our innovation subcommittee is very aware of it. So there's going to be no restrictions there. All the exec are absolutely on board. SITE LEAD*
**Seniority of economists**	*We were thinking anyone in the team could have been the economist that's going into these organisations. But the more we've got into it and the more we've understood from the sites potentially what it is that we could be doing, the more it became important that the outward-facing economist needs to have a particular set of skills. I think it's a combination of the soft skills around the relationship stuff, and being able to listen to somebody, extract a brief, and being able to apply, then the economics background that we have and bringing that critical reasoning to that other person's problem. eE 2*
**Appointment of site lead**	*All of my interactions were via [SITE LEAD]…Because, just from a pragmatic point of view, the decision-making that happens in the organisation, you do need the authority behind it. eE 1*

**Table 4:** Summary of embedding phase process facilitators and barriers

PROCESS		QUOTE
**Facilitators**	Physical location of economist in an open plan office in the ‘noisy’ accessible section	*Yes, so that actually does help things because there’s a lot of flow of information and there’s a lot of things that do get around really quickly, which is a real advantage.* PHN PARTICIPANT 11
	Administrative support from economists’ organisation to book formal meeting requests	*…asking [executive assistant] at HMRI to coordinate these meetings - have provided her email to [site lead] so that these can be arranged and coordinated by one person so everyone is on the same page.* eE 1
	Identification of existing in-house expertise	*We already had an economist that we didn’t know about … so she’s already got a real interest and expertise in that area, so that was really handy to have someone who could put her hand up and say, “Great. This is terrific and I’m more than available to continue to push those things.”* PHN PARTICIPANT 11
**Barriers**	Depth of embedding	*…you can't just eliminate all the other things that you have on your books. So we don't have the luxury of being able to just be focused mentally on what's in front of us for this. There's a whole heap of noise and movement with all of the other projects at different stages of completion that are in the background, that get sidelined …everybody's juggling multiple demands.* eE 2
	The need for a communication and visibility strategy	*It seemed a little bit confusing in terms of what the embedded Economist was here to do, and for who. I think we were getting some mixed messages from the executive about that. You know, so we were told that there were specific programs that had been identified and that’s what would be worked on. But then we were also told, you know, at any time go and have a chat and see what we can do. And that probably created a little bit of conflict.* PHN PARTICIPANT 14
	The need for an exit strategy	*The ugly is that projects and questions are coming to me now that I’ve got a very limited time to really to properly answer.* eE 1
	Length of embedding	*My only criticism was that I would have liked to have seen him around for more than three months. So, I felt that three months was too short. I think he should have been with us for at least six months or probably 12 months. PHN PARTICIPANT 1*

**Table 5:** Summary of planning and embedding phase contextual facilitators and barriers

CONTEXTUAL		QUOTES
**Facilitators**	Organisational form, size and culture	*…it’s also a smaller organisation, so … relationships with people are easier to form… that those that are employed with the PHN are passionate about primary health care… so that makes a real difference in terms of the culture.* SITE LEAD
	Relevance to organisational function	*You know, obviously people in health planning and, you know, other teams, probably that’s a very direct correlation between what the embedded Economist does, and their work.* PHN PARTICIPANT 1
	Acknowledgment of the need to upskill in economic evaluation	*…it's a clear gap in our skillset, and we need to be in a position to improve our decision-making particularly around economic factors.* PHN PARTICIPANT 2
	Highly motivated staff	*The staff are really keen to extend their own knowledge and they see this as a great opportunity* SITE LEAD
	Stage of organisational evolution	*I think to some extent we’ve been in a very rigorous establishment process as the PHN for a few years now, attempting to commission services and support, improvement in a range of services [but] we haven’t necessarily had the capacity and capability to demonstrate our impact sufficiently. …it’s part of our evolution and maturity.* PHN PARTICIPANT 4
	Modest expectations and an openness to learning	*We'd like some clarity around some of our processes and procedures, certainly in the commissioning space… as well as our health planning processes, we're really keen on that… Improving, we want improvement. We're willing to sort of put our hand up and say "We're not doing this very well." …So we want to be able to improve our processes and our evaluation and our commissioning area… So we want the economist to come up with some ways - or at least do some research - around how we can do that, or how we can capitalise on what we're already doing.* SITE LEAD
**Barriers**	Limited staff time	*…barriers are around your own priorities and what you’ve got going on at the time …I just wish I’d tapped into it more. I wish I’d had more involvement…but I think that’s just how it’s unfolded PHN PARTICIPANT 10*
	Geography	*I think Newcastle staff have potentially gained more from it because of the, you know, bums on seats approach. That always works that way, that there’s – if there’s somebody in passing who you’re having a chat to in the kitchen, who might say what are you doing this week, and have those sorts of even unplanned interactions.* *PHN PARTICIPANT 9*

#### Process facilitators and barriers

Three process issues facilitated the *planning phase.* The first was Board and active executive sponsorship within the HNECCPHN. The second was the seniority of the lead economist and applied experience of all the support economists. More than 20 years of applied experience working with health services, and an understanding of the regulatory context in which the PHN operated were considered crucial to encourage executive teams to consider new ways of thinking and working. Thirdly, the eE Program was facilitated by the appointment of a site lead, a PHN senior executive who ‘concierged’ the program from within the organisation, liaising with the program manager and acting as a gateway for staff to access the economists.

Three process issues facilitated the *embedding phase:* the physical location of the economist in an open-plan office; a single contact point and administrative support to book formal meeting requests; and identification of existing in-house expertise.

Process challenges in the embedded phase included: depth of embedding (economist time available per week); the need for a communication and visibility strategy; the need for an exit strategy; and the overall length of embedding period. The economist embedded for 328.7 hours - approximately 22 hours per week. This partially embedded model was implemented due to the available project resources. It meant however that the economist was not fully relieved from their everyday jobs and was constrained by competing interests and demands. Despite this, the economist was available outside of their physically-embedded time by telephone and via virtual meetings.

The first two weeks of embedding resulted in less engagement than expected, with only 20 staff members engaging over this period. A more detailed communication strategy was then developed consisting of: three morning teas (one-face-to-face and two conducted via Skype with satellite offices) where the lead economist explained the program to managers; a ‘pitch’ emailed by the site lead to managers asking for project nominations to be ranked in order of organisational priority by executive; and a presentation by the lead economist at the annual ‘All Staff Day’ to increase program visibility and foster staff engagement. Given the success of the lead economist's presentation at the ‘All Staff Day’ and with the benefit of hindsight, the site lead thought earlier communication, engaging all staff, and clarifying the process for engaging with the economist to ensure a strategic approach to this limited resource, during the planning and embedding phases would have been beneficial. Staff also wanted improved communication throughout the embedded phase, including about what other projects the economist was working on. Overall, embedded research takes time to build visibility and momentum, timeframes may need to be adjusted to accommodate for this and staff need to be made aware that not everyone will have access, particularly when the economist was only intended to be placed at the PHN for a limited period.

Despite a slow start, a crescendo of projects presented toward the end of the embedded period, predominantly as a result of visiting a satellite site for the first time during the last week of embedding, resulting in ongoing remote work. The desirability of developing an exit strategy and moderate expectations about how much work can be done and when the work will end earlier in the program was identified.

The embedded component of the HNECCPHN project was scheduled for three months but actually went over time by approximately three weeks. Three months was perceived by all participants as the shortest possible timeframe, with six months cited as more realistic, as projects presented late into the embedded phase, right up to the last day of embedding. A longer lead time for the economist to immerse within the organisation and identify appropriate projects and priorities would have been beneficial.

These process issues are all interrelated as they represent consequences of limited economist time *and* eventual high demand for services. For instance, a slightly slow start may not have been identified as an issue with a different schedule or if the engagement was not perceived as very high value.

### Contextual facilitators and barriers

A number of organisational characteristics impacted positively on the *planning* and *embedding* phases of the HNECCPHN program. The PHN’s medium size (approximately 100 full-time equivalent [FTE]) cultivated an enthusiastic and receptive culture where employees would easily see the benefit and impact of the eE Program. These factors made it feasible for the eE Program to achieve broad organisational reach.

Relevance of the eE Program to organisational function was another facilitator. Given the focus of the eE Program was on upskilling participants in economic evaluation, its relevance to commissioning was easily understood. Senior executives and staff recognised the lack of skills and capability in relation to economic evaluation and were motivated to take the opportunity the eE Program presented.

The eE Program therefore came along at a time that aligned with the PHN’s stage of organisational evolution. The organisation was moving from an establishment phase to attempting to commission services, support improvement in a range of services and demonstrate impact. It was hoped the eE Program would provide tools and training to inform more systematic or rigorous way of making decisions about planning and commissioning.

Staff’s limited time impacted on engaging with the economists, with some participants expressing regret at the end of the program that they had not had time to engage more.

Geography impacted negatively on the eE Program at HNECCPHN. Embedding at least partially face-to-face onsite was considered essential to the program by all participants, predominantly because of the opportunities this presented for quick, unplanned interactions and real-time feedback. There was the perception that staff in the Newcastle office benefited more from the project than staff in other offices because of the greater face-to-face contact Table 5.

### Relational facilitators and barriers

The following three subsections set out the themes and sub-themes that emerged from data coded under ‘relationality and engagement.’ This code collected data that addressed: how and why engagement occurred or did not occur; and what relational processes, mechanisms, attributes and skills were required for engagement to occur.

Senior executives (*N*=3) and staff participants (*N*=12) expressed a number of positive attributes held by the economist that they perceived as facilitative of the program, as set out in Table 6. Economists (*N*=2) and PHN executives (*N*=2) and staff (*N*=16) provided insights on the ways of working.

**Table 6:** Summary of relational facilitators

RELATIONAL		QUOTE
**Relationship building**	Pre-existing relationship	*We had established and good relationships … so we are already at first base eE 1*
**Economists’ attributes as perceived by staff participants**	Intellect and knowledge of subject matter	*Just a really smart head... he knows his stuff. PHN PARTICIPANT 5*
	Ability to clarify and demystify difficult concepts	*[eE] has a really nice way of communicating those things in a very – I wouldn’t say simplistic, but it is quite simple and it’s not onerous for people who don’t have that kind of evaluation perspective, or don’t have that experience or background in thinking about evaluation or thinking about value-based healthcare or those sorts of things. PHN PARTICIPANT 11*
	Ability to make subject matter interesting	*He makes health economics sound extremely interesting. It was really inspiring listening to him…He’s really touched some people who would … have heard the words health economics and run in the other direction PHN PARTICIPANT 20*
	Responsive, engaging, approachable, and encouraging communication style	*Well obviously good and positive was [eE’s] willingness and ability to jump into something very quickly in a very short turnaround time and give us some feedback, which was great... So he’s quite responsive. PHN PARTICIPANT 1* *Having an economist who can really engage well with staff was a key enabler. Not – not all economists can do that, so that was – that was a key enabler. PHN PARTICIPANT 4* *He’s been very approachable and has been very open to having discussions with anybody and everybody. PHN PARTICIPANT 9* *It comes back to the response that she received, and the encouragement she received helped her go further. SITE LEAD*
	Treating staff as equals	*He can relate to anyone at their level. He doesn’t come across as being superior PHN PARTICIPANT 15*
	Displaying genuine interest in participants’ work	*I think too that [eE] is able to demonstrate an actual interest in the work that people are doing and has been quite vocal about the value that he’s getting out of the experience. I think that really has resonated with people so they don’t feel like – I guess there was a risk that we could have been … treated like lab rats in some ways, but that’s definitely not the experience. PHN PARTICIPANT 3*
	Incisive and gently directive	*I've really enjoyed and really appreciated the take-charge and the authority. Not in an overpowering way at all, just probably his comfort in moving things along quickly…So his ability to be able to give me some answers and some direction incredibly quickly was incredibly helpful. PHN PARTICIPANT 12*
	Relevant	*He’s been well received. I think because even on a personal note he’s just really interested in the topic, but he’s also interested in making it relevant for us. PHN PARTICIPANT 17*
	Adaptive	*…he was adaptive. So what I mean by that is he – he didn’t take a rigid university based or academic style health economic approach. He listened to people then adapted the approach to impact assessment or health economic support to the need of the – the program, project or initiative. Which is different from the traditional health economic approach where you pull your academically developed resource, or whatever it is, off the shelf, and fit – try to fit the initiative into that. So it’s a different – it’s a different approach, and to me that was a key part of the success to be honest, in [eE’s] style. PHN PARTICIPANT 4*
**Economists’ attributes as perceived by economists**	A quick thinking, solution-based, confident and facilitative approach	*The need to think on your feet super quickly. You’ve got … an hour and … your mind’s got to be going through what potential solutions might be. You’ve got to be confident enough to say, if there isn’t a solution, ‘you’re going to have to wait and I’m going to have to come back to you’ … And there’s been times that people have told me their problems and I actually know, that’s not my skill set, ‘So you need to talk to [someone else] …if I said something I’d be guessing.’ And you need the confidence to be able to say that. eE 1*
**Ways of working**	Coaching	*It is about that mentoring, coaching approach … [the eE] just takes people through step by step, doesn’t land a whole lot of information on somebody’s desk and expect them to digest it. He sits down with you, with the information in front and goes through it. So not a dump and run, a gentle reading. PHN PARTICIPANT 20* *I want [LEAD and SUPPORT ECONOMISTS] to support me rather than them do all the work and then just hand me a cost model at the end because then I don’t learn anything PHN PARTICIPANT 18* *Understanding that what health services need and what the academic institutions think they need are really two different things. That it can be quite insulting to them to have an academic tell them, ‘There's a better of doing this you know.’…The attitude I think that's going to be important is, ‘Right, you've got a problem, let's do the best we can to get this problem sorted.’ Not telling them how to reshape their business model…. eE 1*
	Bi-directional learning	*…we're learning as we do this program… for my personal development, it has been a river of gold…When I went into the PHN it was a … a new learning for me that we were not as applied as I thought we were…people were asking me issues around evaluation and economic evaluation that were almost like first steps that we tend to ignore…normally we would have just launched straight into, ‘Oh, that’s the project, you want an evaluation of that, this is what the evaluation’s going to look like.’ What we were missing previously is that understanding that they needed help with that background work of ‘what are the pathways to determining you actually have a project that even warrants an economic evaluation.’ eE 1* *It was really interesting to find out more about health care systems at the local level. So, it’s been a good satisfying learning experience for me… So, I’m a better health economist for having done those jobs. I can certainly say that. eE 3* *In a normal situation, usually if I do a job for somebody … they won’t learn much about what I do. …. If they’ve never seen a cost analysis before, of course they’ll see it for the first time and I’ll talk about how I did it. But they won’t get a chance to work with me. In this case I actually encouraged [DE-IDENTIFIED]… to make changes in the spreadsheet…we worked together closely… It was more hands on in this case. So, it wasn’t like a normal job. It was very much one where it was set up on the initial understanding of the closer working relationship with a practical aspect. eE3*

These relational facilitators enabled the economist to provide tailored support and capacity building to facilitate the application of relevant tools and approaches to specific problems within the health service. Participants (*N*=12) in four of the six projects undertaken with the economist commented on this coaching approach. ‘Coaching’ was characterised by the lead economist as problem solving rather than imposing a solution.

Post embedding, the economist’s willingness to continue coaching three staff members, impacted positively on the program in that it enabled further capacity building to occur and ensured the trust built throughout the embedded phase was not broken by abandonment at the end of the program timeframe. During this phase ‘coaching’ was less intense. The eE’s field diary reveals ongoing sporadic email and Zoom contact about two projects over approximately two- and one-half months and email contact for approximately one year post-embedding.

This way of working was described by one economist as different from their usual way of work in that closer relationships were formed facilitating greater learning for both the economists and PHN participants. Coaching was underpinned by bi-directional knowledge exchange, with the economists learning as much as staff participants.

## Discussion

Overall, our results confirm the commentary available on embedded research with similarities between our process, contextual and relational facilitators, challenges and barriers, to the high-level ones identified by previous researchers examining the implementation of embedded models in health care [12, 13, 18, 19, 22, 25]. Particular practical lessons that should be considered when implementing future embedded research are catalogued below.

### Process factors

We confirmed the importance of a number of previously suggested process facilitators including the need to appoint site leads [22, 25]. Whilst our process for communication and engagement did result in securing executive engagement and the identification of a champion in the form of a trained economist, difficulty came in explaining how the economist would work and managing expectations about level of embeddedness and chiefly, how the eE would leave the site. Engagement should have occurred earlier and prior to embedding, in a longer designated planning phase [18, 19, 22]. The development of an operational plan helped with scope, but the volume of work in the available time and exit from the site were difficult to manage – especially considering the longer than expected lead time to co-produce the scope of work. We all had ‘little experience of the roles and therefore it took time and learning from all parties to embed in the role’ [24]. However, we advocate for the need for any planning phase to ensure program flexibility. Once health services identify the problems they wish to address, the program needs to be able to pivot to provide whatever skills are required. This might mean there is a need to find another researcher with a particular expertise, for example, statistical modelling.

Our program funding extended only to a part-time embedded researcher for three months. The time it took to gain momentum, the need for work to be conducted post-embedding and the economists’ struggles with competing work interests suggests more secure funding to allow flexibility in length and depth of embedding would have been preferable [18, 22, 24, 25].

Co-located workdays (including having a desk, access to IT systems, administrative support, communal areas and meeting rooms) enabled staff to seek informal support, which was greatly valued by staff in this particular location. Pain and colleagues suggest, ‘feelings produce impacts produce feelings’: emotional dimensions are not side-effects but are active in generating impact [27]. The warm relationships developed were central to program success. Establishing relationships requires a large investment at the front end of a program necessitating a ‘high level of physical presence and face-to-face interactions.’ [13]. Once established, these relationship may be maintained virtually, however some ongoing face to face contact is necessary [13]. Lack of co-location was challenging for the geographically dispersed PHN sites that did not have face-to-face access, confirming Vindrola-Padros’ (2019) research [13].

### Contextual factors

The form, size and culture of the site all contributed positively to the program [18]. The PHN is a medium size organisation (approx. 100 FTE) – so a small number of economists could feasibly effect change, with new conversations about evaluation documented as occurring widely. It is a relatively new organisation (established 2015), open to learning and actively seeking to improve the way it works and develop better processes. The PHN committed to working with the economists to upskill staff in economic evaluation, a skill deemed necessary and lacking by both senior executives and other staff to improve decision-making. The economists approached the embedded phase well informed of the organisational context and with a willingness to be responsive and adapt to organisational needs. The PHN itself was open and responsive to the eE Program. Staff were highly motivated to increase their skills in economic evaluation to improve commissioning and ‘make a difference’ [22]. The subject matter was seen as responsive and tailored to organisational needs as PHN funders are placing increasing emphasis on the need to demonstrate value (external context) and much of the co-designed work had a focus on impact assessment, highly relevant for all the work of a commissioning organisation [19, 23].

### Relational factors

The literature acknowledges the importance of embedded researchers having social and interpersonal skills as well as technical or topic specific expertise. Desirable interpersonal skills include inquisitiveness, receptiveness and enthusiasm as well as communication skills [18, 22, 24]. Our findings are confirmatory - embedded researcher skills and attributes are pivotal in establishing the way of working or interactions during the embedded period, which is in turn pivotal in ensuring success.

However, our research goes further and adds to the importance of relational issues in embedded research. The greatest facilitator for this program was the way of working, the ‘coaching’ or ‘slow science’ [18, 29] approach facilitated by the economists. Whilst previous authors have highlighted a variety of ways of working based on: degrees of objectivity and levels of embeddedness [18, 33, 34]; the ‘role domains’ of knowledge brokering [14, 35] and ‘mechanisms of collaboration for co-production’ [36]; we argue that embedded research will benefit from viewing the work done or interactions through an additional lens of embedded processes as ‘slow science’ [28, 29].

In line with our philosophic commitment to slow science [28], these results build on that thinking by unpacking how a process of capacity building can occur during embedding. Building capability in health economics, or any other expert skill within health or commissioning organisations, calls for a ‘re-invention’ of health service research: moving from the pursuit of grand scientific findings that apply to all services everywhere, to a more nuanced combination of understanding local context and needs, workforce capacity building, design of realistic plans of action, and programs of investigation targeting priority problems of the health service. Instead of bypassing complex challenges in order to adhere to theoretical-methodological strictures, this health service research re-invention ensures we take local complexity as our primary point of departure. However this only becomes possible when we engage healthcare staff as co-researchers and prioritise practical achievement and future-facing learning over the production of discipline-specific knowledge and method-delimited evidence [28].

### Strengths and limitations

These results must be considered in light of the following limitations: the eE Program embedded a particular skill set – health economics – in a particular context – a Primary Health Network located in regional Australia. The evaluation of future Local Health District (care provider) sites at which the economist will embed within the NSW Regional Health Partners' footprint may produce quite different results. This is a small scale, qualitative case study. Whilst all program participants from this site were approached to participate in the evaluation, participants self-selected which increases the risk of bias. The evaluation was conducted by a social scientist who also occupied the dual role of program manager. However, to ensure the research remained theoretically and methodologically sound, the embedded Economist program was overseen by the eE Social Science Research Committee.

## Conclusion

This article demonstrates the previously reported evaluation components of the study protocol [1] to be effective in identifying barriers and facilitators to embedded research. Our evaluation contributes to the empirical evidence on embedded research in three main ways. First, it examines embedded researchers in a previously unexplored context: a regional Australian primary health commissioning network. Secondly, it describes a detailed design/schema for an embedded program and ideas for improvement that can be adapted by others. Thirdly, it adds to the importance of relational issues in embedded research: the greatest facilitator for this program was the way of working, the coaching or ‘slow science’ approach employed by the economists.

Despite its stretched resources, this embedded research project had impressive outcomes. The organisational outputs, in the main, will not be reflected by academic outputs. Hopefully, this case study is the start of a journey where continued funded engagement will increase HNECCPHN staff skills and the development of research endeavors that are democratically designed and undertaken and also contribute to the academic literature. Elsewhere we outlined the view that healthcare research warrants adopting a more local, contextualised and practical approach in order to tackle health service complexity with requisite levels of fine-grained sensitivity and local relevance [28] .

Overall, this project worked because the organisation was highly receptive and the skills offered were a perfect fit for a commissioning; because of the democratic stance taken by the economists (‘also learning’) and the economists' efforts to encourage the accessibility of their tools and techniques (‘it’s not rocket science’) and; finally, due to the warmth and enthusiasm all parties brought to the engagement.

## Supplementary Information

**Additional file 1.**

**Additional file 2.**

**Additional file 3.**

## Data Availability

The datasets generated and analysed during the current study are not publicly available due to the small sample size and size of the participating organisation. The researchers did not receive consent from participants to make data publicly available, as participants required anonymity to ensure they spoke freely about their organisation and the program. To access available data sets, please contact Dr Lisa McFayden, Lisa.mcfayden@health.nsw.gov.au

## Abbreviations

ComPrac: Community of Practice
eE: embedded Economist
HMRI: Hunter Medical Research Institute
HNECCPHN: Hunter New England Central Coast Primary Health Network
IT: Information Technology
LHD: Local Health District
NSWRHP: New South Wales Regional Health Partners
PHN: Primary Health Network
