# Supplementary material for: Relational aspects of building capacity in economic evaluation in an Australian Primary Health Network using an embedded researcher approach
Source: BMC Health Serv Res. 2022 Jun 22;22:813. doi: 10.1186/s12913-022-08208-7 (PMC9219146; doi:10.1186/s12913-022-08208-7)
Supplement: Supplementary file 1 — Additional file 1. [file 12913_2022_8208_MOESM1_ESM.docx]

**Additional File 1 Overview of the embedded program and participants as delivered at the HNECCPHN**

| PHASE | TIMING | PLANNED ACTIVITIES | PARTICIPANTS | PURPOSE | ACTUAL ACTIVITIES |
| --- | --- | --- | --- | --- | --- |
| PLANNING | 6 months prior | Project briefing to all sites at NSWRHP Translational Committee meeting | NSWRHP Translational Committee members including PHN Site Lead | To introduce the program and understand the context, priorities and expectations of the NSWRHP partner sites and engage the sites as participants | As per plan |
|  | 1 month prior | Formation of Program Advisory Committee(With meeting schedule including 2 meetings during the embedded phase) | eE Steering Committee made up of external chair, site representatives, external economist, consumer representatives, the program lead, lead economist, and program manager/social scientist as well as two social science professors (forming a social science sub-committee) | To strategically advise on the program protocol and risk management and to form a sub-committee of senior social scientists to advise on program evaluation | As per plan |
|  |  | Introductory meeting PHN senior executive, economist and researchers | Program leadLead economistSupport economistProgram manager/social scientistSite leadChief executive1 additional executive | To introduce the program and understand the context, priorities and expectations of the PHN | As per planExecutive were very supportive and had few preconceptions of how the program would evolve |
|  | 3 October 2019 | Presentation of introductory seminar on health economics and the program, covering:Core concepts of health technologies and health technology lifecycleAn economic perspective on some problems in healthcareA summary of the range of possible economic evaluations AND the questions they can answerFollowed by discussion on:How could health economics be useful to the PHN?If the PHN had specific projects in mindAt the end of the embedded economist program, what would “success” of this program look like?(2 hours) | PHN seminar attendees (N=10)4 senior executive2 health analysts3 health planners/managers1 commissioning manager | To begin capacity building and introduce the program as well as the site lead, economists and social scientist to a broader group of staffTo prioritise areas the economist might focus on | All elements of the presentation were delivered without modificationThe discussion questions prompted a brainstorming session on what the economist could work on. The site lead wound the seminar up by stating the next steps would be for her to collect ideas and feedback from participants and discuss with economist when he embedded |
|  | November 2019 | Drafting operational planThe lead economist and an additional HMRI economist developed a generic draft operational plan to be tailored to the PHN’s specific needs during the embedded component. This operational plan was designed to provide clarity over the delivery and evaluation of the embedded component as well as set out expectations of what sites are to provide to enable the embedded Economist Program to be successful, and the requirements of the embedded economist to optimise benefit of the program to health services. The plan contained the following sections:Seminar on health economic evaluationsGuidelines for initial meetings with health servicesProtocol for the embedded economist placementEvaluating the embedded Economist ProgramHealth service expectationsTimelines for the embedded Economist ProgramEmployment considerationsDebrief – post placement | Lead economistProgram manager/social scientistSite lead | To provide clarity over the delivery and evaluation of the embedded component as well as set out expectations | The plan was an effective starting point for all parties to discuss how embedding would occur |
| EMBEDDING | 16 October 2019 - 29 February 2019 (with a 1 month hiatus for Christmas and New Year) | Lead economist embeds at PHN Newcastle office 2-3 days per week in person and via email and telephone 1 day a week. Support economists from HMRI work on projects as directed by the lead economist. | Lead economist and 4 support economists | To build capacity in economic evaluation | The lead and other HMRI economist time equated to 328.7 hours during the embedded phase or approximately 22 hours per week.There was a total of 233 engagements between the economists and staff during the embedded phase, with 15.3 average engagements per week, and a median number of 7 engagements per week. (N.B. some staff engaged more than once, accordingly numbers represent episodes of engagement).The 2 satellite sites were only visited once each, because of program resources. Lack of a full-time face-to-face presence was supplemented at ALL sites, including Newcastle with Skype, Zoom and email.PHN staff wanted a combination of advice about incorporating evaluation into ‘business as usual’ as well as training in basics such as: costing a model of care; how to do an impact assessment; what outcomes are important etc. Impact assessment was introduced using the Framework to Assess the Impact from Translational health research (FAIT).* |
|  | Engaging25th October 2019 -4th November 2019 | Presentation by lead economist at annual all staff forum focused on how staff can include health economics in their everyday workGastro-diplomacy eventA morning tea and/or a meet and greet/pitch10am in person at PHN Newcastle offices10.30am via Skype to Erina office11am via Skype to Tamworth Office | Lead economist and ALL PHN staffLead economist and program manager/social scientist, 36 PHN staff across 3 site offices | To improve engagement of staff with the economist | These engagement events were an addition to the operational plan, co-designed by the lead and lead economist after less than expected engagement in the first 2 weeks of embedding. Both were successful, resulting in greater engagement post events. An engagement strategy should have been considered in operational plan. |
|  | Celebrating | Website | Lead economistProject leadSite communications officerSite leadProgram manager | To celebrate the achievements of the site and create broader program visibility for future sites, the funder and interested researchers | A website housing program resources including one video case study was developed, see: <https://embeddedeconomist.com.au> |
| POST EMBEDDING | 4th March 2020(Week following embedded economist leaving site) | Exit meeting - 10am-11am via telephone | Lead economistSupport economistSite lead | To discuss its achievements and lessons learnt and sustainability of capacity building | As per plan |
|  | February 2020 - February 2021 | Virtual contact with project lead to finalise projects (time not collected) | Lead economist3 site participants | To finalise projects | The site wanted ongoing support and access to a health economist. The lead economist received requests for support up to 12 months post placement. This included mentoring for PHN staff to complete their own cost-benefit model. The embedded economist’s field diary reveals the ongoing contact took the form of email and Zoom contact sporadically over the 12 months post-placement, leading to the conclusion that there is a need to build in an exit strategy and ‘tailing-off’ phase. |

* Searles A, Doran C, Attia J, Knight D, Wiggers J, Deeming S, et al. An approach to measuring and encouraging research translation and research impact. Health Res. Policy Syst. 2016;14(1): 1-13.
