# Supplementary material for: Relational aspects of building capacity in economic evaluation in an Australian Primary Health Network using an embedded researcher approach
Source: BMC Health Serv Res. 2022 Jun 22;22:813. doi: 10.1186/s12913-022-08208-7 (PMC9219146; doi:10.1186/s12913-022-08208-7)
Supplement: Supplementary file 2 — Additional file 2. [file 12913_2022_8208_MOESM2_ESM.docx]

Additional File 2 Number of engagements and type and subject of activity

| WEEK | NUMBER OF ENGAGEMENTS | TYPE OF ACTIVITY & SUBJECT MATTER |
| --- | --- | --- |
| 1 | 4 | 1 telephone meeting with site lead to orient the embedded economist  3 face-to-face approaches by staff, about developing a business case, conducting a cost study and impact assessment |
| 2 | 16 | 1 face-to-face meeting with 5 senior executive to confirm a focus by the embedded economist on impact assessment  11 face-to-face approaches by staff about a variety of issues ranging from program evaluation generally through to how to build evaluation into commissioning contract, impact assessment, costing studies, dynamic modelling |
| 3 | 7 | 1 Skype meeting about a potential project consisting of an evaluation design and costing study of the Medical Practice Assistance Program  1 face-to-face approach by an in-house existing economist wanting to further build capacity in costing  1 face-to-face approach by 2 members of staff about evaluating Primary Health Network (PHN) risk strategies  3 face-to-face approaches about impact assessment |
| 4 | 38 | 37 attendees at introductory morning teas (10 face-to-face in Newcastle; 16 via Skype in Erina; and 11 via Skype in Tamworth)  1 approach about evaluating telehealth |
| 5 | 11 | 7 attendees at 3 hour face-to-face impact assessment workshop at Erina site  1 Skype meeting with staff member from Tamworth about potential project consisting of an evaluation design and costing study of fracture prevention program in a general practitioner (GP) setting  1 meeting with 3 staff members from the PHN Impact Assessment Working Group |
| 6 | 2 | 1 face-to-face meeting with staff member from Newcastle about evaluating a GP model of care  1 Skype meeting with staff member from Erina to confirm progressing on the evaluation design and costing study for the Diabetes Alliance Project |
| 7 | 112 | Presentation on economic evaluation at PHN all staff day |
| 8 | 6 | 1 face-to-face meeting with 3 members of staff and a supporting economist to progress work on the Diabetes Alliance evaluation and costing study  1 Zoom capacity building session with in-house economist on costing techniques  1 face-to-face capacity building session with staff member on impact assessment and logic models  1 telephone capacity building session with staff member on impact assessment and logic models |
| 9 | 4 | Several virtual communications (email and Zoom) with 1 member of staff to draft cost model for Diabetes Alliance Project  1 face-to-face capacity building meeting to provide overview of the Diabetes Alliance costing model to in-house economist  1 workshop on impact assessment presented to 2 members of staff at their request |
| 10 | 1 | Face-to-face meeting to discuss how to approach evaluating the PHN Risk Assessment Framework |
| 11 | 6 | Several virtual and face-to-face meetings with 2 members of staff to progress the evaluation of the Diabetes Alliance Project, including developing a business case  1 virtual meeting with 1 member of staff to discuss evaluation of 2 new projects: the Rural Communities Project and the Trusted Advocate Project  1 face-to-face meeting with 1 member of staff to draft logic models for the evaluation of the PHN Risk Assessment Framework  2 face-to-face approaches about general evaluation questions |
| 12 | 7 | 1 face-to-face capacity building session with 3 members of staff with support economist presenting the final costing model for the Diabetes Alliance Project evaluation and its application to the Fracture Prevention Program evaluation  1 face-to-face advice session with 2 members of staff on how to complete a logic model for the Fracture Prevention Program  1 face-to-face capacity building session with in-house economist on taking over costing PHN projects after the embedded economist leaves  1 face-to-face meeting with site lead and program manager about the need for an exit strategy |
| 13 | 4 | Several emails to progress the Medical Assistance Practice Program with one member of staff  1 face-to-face approach to present the Medical Assistance Practice Program evaluation at a conference  1 face-to-face approach to provide assistance on the economic evaluation section of a grant application  1 approach seeking general evaluation advice |
| 14 | 7 | 1 face-to-face meeting with PHN chief executive officer to discuss next steps after the eE Program to ensure sustainability  1 telephone call from a senior executive offering the embedded economist a permanent position at the PHN  1 face-to-face meeting at the local health district in Gosford with PHN staff member to present the Diabetes Alliance results  1 virtual meeting with 2 staff members, the lead economist, the support economist and an additional 2 support economists from HMRI to discuss the Medical Practice Assistance Program evaluation to seek further funding to conduct the evaluation plan after the embedded economist leaves  1 face-to-face meeting with 2 staff members to discuss evaluation of PHN commissioning contracts |
| 15 | 8 | 1 2-day face-to-face visit to satellite site (Tamworth), where 8 staff were engaged via face-to-face meetings seeking further advice to progress evaluation of 3 projects: The Rural Communities Project; the Trusted Advocate Project; the Fracture Prevention Project; as well a meeting to discuss a project not previously raised with the embedded economist: evaluating a cardiac model of care in a GP setting.  1 meeting with site lead and program manager to discuss how the embedded economist would assist in winding up work post-embedding |
| TOTAL | **233** | **Average engagements per week: 15.3**  **Median engagements per week: 7** |
