# Supplementary material for: Relational aspects of building capacity in economic evaluation in an Australian Primary Health Network using an embedded researcher approach
Source: BMC Health Serv Res. 2022 Jun 22;22:813. doi: 10.1186/s12913-022-08208-7 (PMC9219146; doi:10.1186/s12913-022-08208-7)
Supplement: Supplementary file 3 — Additional file 3. [file 12913_2022_8208_MOESM3_ESM.docx]

**Additional File 3 Summary of the embedded Economist (eE) HNECCPHN Projects**

| PROJECT | BACKGROUND | NEED | OBJECTIVE | OUTPUTS TO DATE |
| --- | --- | --- | --- | --- |
| Upskilling General Practitioner (GP) Administrative Staff Impact Assessment  Pilot Project [Medical Practice Assistant (MPA) Project] | This project addressed national priorities in the rural health workforce by upskilling GP administrative staff in a number of non-invasive procedures | A business case and an impact assessment (including a cost consequence component) was needed to examine the relative costs and benefits associated with program impacts, such as the time saved by registered nurses on tasks which are now being performed by MPAs) who have a lower salary, and changes in work satisfaction across different job roles | To upskill relevant PHN staff in impact assessment and costing  To determine the best method of evaluation and draft evaluation plan including methods | - Funding secured from NSW Regional Health Partners (NSWRHP) to conduct impact assessment on the MPA Project - Training Primary Health Network (PHN) staff in impact assessment - Presentation of a draft protocol at the Hunter New England Central Coast PHN (HNECCPHN) MPA conference June 2021 confirmed - Program logic for an impact assessment drafted - Payback metrics identified which cover the domains of research impacts of the MPA Project - Cost model development commenced |
| Central Coast Diabetes Alliance Cost Study | Modifications in diabetes care service delivery were made on the Central Coast. A feasibility study of the new model of care (case conferencing) was designed and implemented. The study had an evaluation arm based on process and outcome metrics but no costing | How much does it cost to deliver case conferencing (for each stakeholder and overall)?  Is it more expensive than the previous model?  Is it more effective than the previous model? | To upskill relevant PHN staff in costing and cost-consequence evaluations  To add a cost component to the existing evaluation to determine whether this model of care is feasible and cost efficient | - Cost consequence analysis conducted - Business case template developed, tailored to stakeholder needs - Study outcomes reporting template developed - Capacity built on comparing cost to consequence and conducting a feasibility study - Demonstrated net revenue (profit) for one stakeholder, which would not have been identified without cost model - Support and expertise from eE created a substantial and data driven business case - Staff lead has built capacity and utilised skills elsewhere in health services and can now build her own cost models |
| General Practice Fracture Prevention Cost Study  (Study currently on hold due to cost constraints) | A new GP initiative to improve the identification and management of patients with osteoporosis in general practice and reduce the number of re-fractures in patients with osteoporosis who have experienced previous minimal trauma fractures had been developed | How much does it cost?  What outcomes need to be measured to evaluate the project? | - To design an appropriate cost / cost consequence model - To upskill a PHN staff member to conduct the costings and develop and collect appropriate outcome measures to support program evaluation | - Upskilling PHN member to conduct the costing and evaluation - Co-producing a program logic and working cost model template - Enhanced connections with external experts in evaluation, economics and statistics - Enhanced internal collaboration between subject matter experts and health planning |
| Healthy Weight Evaluation Project | The increase in the overweight and obese population is a health and strategic concern, with potential impacts on downstream chronic disease. This project pre-dated the eE. The economic evaluation was able to extend in scope due the presence of the eE | Is the Healthy Weight Program cost effective?  Can it be financially viable from a PHN and GP perspective?  How will households engage? | To engage PHN staff in an economic evaluation that would normally have taken place as a standard ‘consultancy’ (i.e. at arms-length from the PHN). The aim was to better engage PHN staff in the *process* of the evaluation  To ensure optimal implementation of this model of care | - The cost model for this project utilised the model developed for the Diabetes Alliance cost study set out above - Economic evaluation, pricing models of the Healthy Weight Program, and design modifications of the program improved effectiveness and efficiency |
| Rural Communities Project and  Trusted Advocate Project | - The Rural Communities Project is a community engagement initiative to assist primary care in areas affected by drought - The Trusted Advocate Project is an intervention in Scone, where the PHN provided training to selected people who were often approached by community members seeking advice/referral (including but not limited to advice on mental health issues) | How can these projects be evaluated and what is their impact? | To build staff capacity to conduct impact assessments on both projects | - Detailed and project specific advice about introducing impact assessment to both programs was provided - Upskilling PHN member to conduct assessments, including but not limited to drafting logic models and assessment tools - Evaluation and impact assessment currently being conducted by PHN staff member |
| Building impact assessment into operational decisions | The PHN increasingly needed to demonstrate ‘value for money’ to government funders | The PHN needs an organisation-wide approach to measure the impact of its programs and build staff capacity in applying the approach | To integrate and build organisational capacity in applying the Framework to Assess the Impact from Translational Health Research – the FAIT Framework [35] | - Staff training on impact assessment via workshops conducted - FAIT applied to a major PHN initiative (Medical Practice Assistant Project – publication of results with PHN co-authorship out for review) |
